# Mitochondrial Function in Enamel Development

**DOI:** 10.3389/fphys.2020.00538

**Published:** 2020-05-29

**Authors:** Veronica Costiniti, Guilherme H. Bomfim, Yi Li, Erna Mitaishvili, Zhi-wei Ye, Jie Zhang, Danyelle M. Townsend, Marta Giacomello, Rodrigo S. Lacruz

**Affiliations:** ^1^College of Dentistry, Department of Molecular Pathobiology, New York University, New York, NY, United States; ^2^Department of Cell and Molecular Pharmacology and Experimental Therapeutics, Medical University of South Carolina, Charleston, SC, United States; ^3^Department of Drug Discovery and Biomedical Sciences, Medical University of South Carolina, Charleston, SC, United States; ^4^Department of Biology, University of Padova, Padua, Italy; ^5^Department of Biomedical Sciences, University of Padova, Padua, Italy

**Keywords:** mitochondria, enamel, ameloblasts, oxidative phosphorylation, redox

## Abstract

Enamel is the most calcified tissue in vertebrates. Enamel formation and mineralization is a two-step process that is mediated by ameloblast cells during their secretory and maturation stages. In these two stages, ameloblasts are characterized by different morphology and function, which is fundamental for proper mineral growth in the extracellular space. Ultrastructural studies have shown that the mitochondria in these cells localize to different subcellular regions in both stages. However, limited knowledge is available on the role/s of mitochondria in enamel formation. To address this issue, we analyzed mitochondrial biogenesis and respiration, as well as the redox status of rat primary enamel cells isolated from the secretory and maturation stages. We show that maturation stage cells have an increased expression of PGC1α, a marker of mitochondrial biogenesis, and of components of the electron transport chain. Oxygen consumption rate (OCR), a proxy for mitochondrial function, showed a significant increase in oxidative phosphorylation during the maturation stage, promoting ATP production. The GSH/GSSG ratio was lower in the maturation stage, indicative of increased oxidation. Because higher oxidative phosphorylation can lead to higher ROS production, we tested if ROS affected the expression of *AmelX* and *Enam* genes that are essential for enamel formation. The ameloblast cell line LS8 treated with H_2_O_2_ to promote ROS elicited significant expression changes in *AmelX* and *Enam*. Our data highlight important metabolic and physiological differences across the two enamel stages, with higher ATP levels in the maturation stage indicative of a higher energy demand. Besides these metabolic shifts, it is likely that the enhanced ETC function results in ROS-mediated transcriptional changes.

## Introduction

Amelogenesis is the biological process that leads to the formation and mineralization of dental enamel by specialized epithelial cells known as ameloblasts (Smith and Nanci, 1995). Amelogenesis can be divided into two main sequential stages: the secretory and maturation stages (Lacruz, 2017). Secretory ameloblasts can reach heights of ∼70 μm with a diameter of ∼4–5 μm, while maturation stage ameloblasts maintain the same diameter but have a decrease in height to ∼40 μm (Smith, 1984; Smith and Nanci, 1995; Lacruz, 2017). During the secretory stage, ameloblasts secrete a limited set of enamel matrix proteins (EMPs). These proteins are involved in building the tissue volume and the enamel crystal organization (Marsland, 1951b; Smith, 1998; Wang et al., 2005; Lacruz et al., 2010). EMPs such as amelogenin (AmelX) and enamelin (Enam) are considered unique products of the ameloblast cells (Smith, 1998; Lacruz et al., 2017). During maturation, the expression of these EMPs declines, and they undergo enhanced proteolysis and endocytosis (Lacruz et al., 2017). Mineralization of the enamel crystals increases concurrent with the increased transport of Ca^2+^ and PO^3–^ enabling a significant expansion in crystal width and thickness (Reith, 1961; Smith, 1998; Lacruz, 2017). During maturation, ameloblasts uniquely cycle from a ruffled-ended border to a smooth-ended morphology (Smith and Nanci, 1989; Smith, 1998).

The biomineralization of enamel relies on an efficient system of ion transport mediated by a battery of pumps, channels and transporters driving the transport of Ca^2+^ and PO^3–^ – the dominant ionic species – as well as HCO^3–^, Cl^–^, K^+^, Na^+^, and Mg^2+^ (Simmer and Fincham, 1995; Smith, 1998; Lacruz et al., 2012a; Lacruz, 2017). The incorporation of ions into the enamel crystals during maturation occurs in parallel with the changes in pH that are strictly regulated to buffer the acidification that results from proton release during mineral buildup (Lacruz et al., 2010, 2012b). Although we continue to increase our understanding of the required protein machinery and processes driving ion transport and mineralization, basic cellular processes linked to- or supporting these functions, remain poorly understood.

Mitochondria are dual membrane bound organelles that are essential in the regulation of cellular metabolism (Lehninger et al., 2003). They are also involved in Ca^2+^ handling and are the center of oxidative phosphorylation via the electron transport chain (Marsland, 1951a) that is responsible for the production of ATP, while also generating reactive oxygen species (ROS) (Rizzuto et al., 2000; Lehninger et al., 2003; Dimmer and Scorrano, 2006). Although some morphological analyses of mitochondria have been reported, the role of mitochondria in enamel development has not been well-characterized to date.

Light and electron microscopy studies since the 1950s (Marsland, 1951a; Watson and Avery, 1954; Pindborg and WeinmannN, 1959; Reith, 1960, 1961, 1963; Jessen, 1968) and stereologic studies (Assis et al., 2003) were performed to visualize mitochondria in enamel cells. These works showed differences in the subcellular localization of mitochondria in secretory and maturation stages (Warshawsky and Smith, 1974; Josephsen and Fejerskov, 1977). In the secretory stage, mitochondria cluster between the nucleus and the proximal or basal pole (Jessen, 1968). Their predominant shape is elongated and their length is variable (Jessen, 1968). Mitochondria in the maturation stage presents two main clusters. A first type show a large cluster found adjacent to the distal ruffled-border (Jessen, 1968; Josephsen and Fejerskov, 1977; Assis et al., 2003), suggesting a potential contribution to the movement of ions from the proximal to the distal pole (Elwood and Bernstein, 1968; Jessen, 1968; Eckstein et al., 2018). These mitochondria appeared as rod-shaped and short (Josephsen and Fejerskov, 1977). A second cluster of mitochondria is found in the proximal pole in the infranuclear region and are mostly spheroidal (Jessen, 1968) while presenting some internal “helices” described as DNA structures (Blecher, 1967; Warshawsky, 1968).

Some reports have indicated that the number of mitochondria increases as the cells transition from the secretory to the maturation stage (Kallenbach, 1970; Assis et al., 2003; Eckstein et al., 2018). It has also been suggested that the size of the mitochondria, itself, is larger in maturation and may reflect a more efficient system in providing higher energy for the cells (Assis et al., 2003). The expression of cytochrome oxidase (CO) activity was found to be upregulated in the maturation stage, with increased CO signals in smooth-ended ameloblasts (Beynon, 1972; Ohshima et al., 1998). These observations prompted suggestions that smooth-ended ameloblasts require more energy to recreate the deep infoldings characteristics of the ruffled-border (Josephsen and Fejerskov, 1977; Assis et al., 2003).

Our own studies also support a role of mitochondria in enamel formation (Eckstein et al., 2017, 2019). Murine models with abnormal store-operated Ca^2+^ entry (SOCE) showed alterations in mitochondrial morphology and function, with increased ROS levels and alterations in the cytoskeleton of the ameloblasts (Eckstein et al., 2017, 2018). Thus, we suggested that mitochondrial generated ROS were important for the integrity of the ameloblasts (Eckstein et al., 2017, 2019).

Given the dearth of knowledge on the role of mitochondria in enamel, the purpose of this study is to characterize whether and how mitochondrial dynamics and function differ in secretory and maturation stage cells. We focused on key markers of mitochondrial physiology: components of the mitochondrial ETC, mitochondrial biogenesis, oxidative phosphorylation, and redox status. We show that mRNA levels of PGC1α, a surrogate for mitochondrial biogenesis, is increased in maturation cells, as well as the amount of mitochondrial DNA. The expression of mitochondrial proteins involved in fusion and fission pathways were decreased. We also found that maturation stage cells show an increase in mitochondrial respiration, ATP production, and increased oxidation. These data highlight the important differences in the metabolic status of secretory and maturation stages of enamel cells, suggesting that mitochondria are a key component in the biology of enamel formation and mineralization.

## Materials and Methods

### Cell Culture

Primary enamel organ (EO) cells were isolated from the lower incisors of Sprague Dawley rats as described (Nurbaeva et al., 2015; Eckstein et al., 2017). The EO was incubated with Liberase (0.25 mg/ml; Roche) for 30 min at 37°C followed by Trypsin for 10 min at 37°C. Cells were then passed through a 70 μm (secretory) and 40 μm (maturation) filters to remove debris and plated onto Cell-Tak (Corning) coated coverslips in X-Vivo15 medium (Lonza) supplemented with 10% FBS and 1% penicillin/streptomycin. Isolated EO cells were used within 24 h after dissection. LS8 cells were grown in Dulbecco’s modified Eagle’s medium (DMEM, Thermo Fisher Scientific) supplemented with 10% FBS and 1% penicillin/streptomycin. 24 h before the experiment, cells were seeded onto plates previously coated with poly-L-Lysine (1:10 in PBS) and then allowed to grow to 70–80% confluence.

### Real Time PCR (RT-qPCR)

Total RNA was isolated using the RNeasy Micro Kit or Mini Kit (Qiagen) as indicated by the manufacturer followed by reverse transcription using the iScript cDNA Synthesis Kit (BioRad). Total DNA was isolated from secretory and maturation enamel organs as previously described (Quiros et al., 2017). For mRNA quantification we used the SsoAdvanced Universal SYBR Green qPCR Supermix (BioRad) and performed the experiments in a CFX Connect Thermocycler (BioRad). All of the primers were used at a concentration of 0.25 nM with *Actin* or *Gapdh* functioning as housekeeping genes. Relative quantification of gene expression was determined by the 2^–ΔΔCT^ method. Supplementary Table 1 lists all primers used.

### Mitochondrial Morphology Analysis

EO cells were isolated and plated onto Cell-Tak (Corning) coated coverslips in X-Vivo15 medium (Lonza) supplemented with 10% FBS and 1% penicillin/streptomycin. After 4 h they were loaded with CellLight Mitochondria-GFP, BacMam 2.0 (Thermo fisher Scientific) according to the manufacturer instructions. Cells were washed and loaded for 30 min with PE anti-rat CD90/mouse CD90.1 (Thy-1.1) (1:500; BioLegend) to identify possible fibroblast contamination. Images were taken using a SP8 confocal microscope (Leica).

### Determination of mtDNA vs. nDNA

We determined changes in mitochondrial DNA (mtDNA) vs. nuclear DNA (nDNA) as a ratio of the two genes as reported (Carabelli et al., 2011; Quiros et al., 2017) by RT-qPCR. The expression of *Rnr2* (16S ribosomal RNA) was used as a gene marker for mtDNA, and *Gapdh* was used as a marker for nDNA.

### Mitochondrial Membrane Potential (MMP)

For analysis of MMP, 10 K cells of secretory and maturation stages were plated per well onto 384-well plates (CellCarrier, PerkinElmer). After 24 h in culture, fluorescent FITC anti-rat CD90/mouse CD90.1 (Thy-1.1) (1:500, 30 min at 37°C; BioLegend) was used to allow to distinguish between fibroblasts and ameloblasts. Cells were rinsed in 10 mm HEPES buffered saline (HBSS buffer, pH 7.4; Thermo Fisher Scientific) and subsequently loaded with 20 nM TMRM in the presence of 1 μM cyclosporine H (30 min at 37°C) and left in the same buffer during image acquisition. Alternate brightfield, digital phase contrast, 488 and TMRM fluorescence (excitation/emission at: 460–490/500–550; 520–550/560–630 nm, respectively) images were acquired every 3 min, using the 20X magnification air objective of the high content screening (HCS) imaging system Operetta^®^ and Harmony^®^ software (PerkinElmer). Cells were treated with oligomycin (1 μM). FCCP (3 μM) was added as a control for mitochondrial depolarization. Analysis was performed by means of Harmony^®^ software (PerkinElmer) as follows. Image segmentation was performed by Region of Interest in the Digital Phase contrast channel. FITC fluorescence intensity was calculated per each individual cell, and background corrected. We considered as enamel cells only cells that did not contain significant fluorescence levels of the fibroblast marker [FITC anti-rat CD90/mouse CD90.1 (Thy-1.1) intensity, background corrected <5]. TMRM Fluorescence intensity, background corrected, was then measured per each region of interest (that is, per each individual cell) and averaged. Number of cells = more than 20 per independent experiment; number of experiments = 3.

### Mitochondrial Respiration

The Mitochondrial Stress Test Kit (Agilent) was used to analyze mitochondrial oxygen consumption in primary EO cells following the manufacturer’s instructions. EO cells were seeded 24 h ahead in a XFe24-well microplate (Agilent) at 4 K cells/well in complete X-Vivo TM15 (10% FBS, 1% penicillin/streptomycin). In parallel, a cartridge plate was hydrated with 1 ml/well XF Calibrant (Agilent) and kept overnight in a non-CO_2_ incubator. The following day XF Base medium (Agilent) with the addition of 1 mM Na-Pyruvate, 2 mM L-Glutamine, 10 mM Glucose at pH 7.4 was prepared. The cells were washed twice with the prepared complete XF medium and refilled with the prepared complete XF medium to a final volume of 500 μl per well. Cells were equilibrated for 1 h in a non-CO_2_ incubator. 1 μM of oligomycin, 1 μM of FCCP and 0.5 μM of Rotenone/Antimycin A were serially added in a Seahorse XFe24 Analyzer. Data were normalized through EVOS FL Auto (Thermo Fisher Scientific) after staining cells with Hoechst (Thermo Fisher Scientific).

### ATP Quantification

ATP was quantified using a luciferase-based kit (Molecular Probes). 10 K of cells were plated in a 96-well opaque white plate with a clear bottom. After 24 h, cells were then permeabilized with cold methanol for 15 min at 4°C. Treatment with ATP (100 μM) and oligomycin (25 μM) for 15 min before methanol addition were used as positive and negative controls, respectively. The standard reaction solution (Molecular Probes) containing the firefly luciferase and D-luciferin was loaded. A Flexstation 3 plate reader (Molecular Devices) was used to read the luminescence at the integration time of 500 ms with normal gain. ATP amount was then measured using a standard curve.

### GSH/GSSG and Thiol Measurements

Total reduced thiols, GSH and GSSG were measured as previously described (Zhang et al., 2018). EO cells were isolated and their extracts were either used directly for total reduced thiol determination with thiol fluorescent probe IV (Millipore), or precipitated by sulfosalicylic acid. The supernatant was neutralized by triethanolamine and divided into two portions. To measure the reduced GSH, one of the supernatant portions was incubated with the thiol fluorescent probe IV (Ex 400 nm/Em 465 nm). For total GSH (GSH + GSSG), the second supernatant portion was incubated with the reduction system (containing NADPH and glutathione reductase) at 37°C for 20 min. The ratio was determined as follows: GSH/GSSG = [GSH]/(([Total GSH] − [GSH])/2).

### ROS Measurements

MitoSOX^TM^ Red Mitochondrial Superoxide Indicator (Thermo Fisher Scientific) was used to measure ROS in LS8 cells. 10 K cells were plated on 96-well black plate with a clear bottom. After 24 h they were loaded for 30 min with MitoSOX (5 μM) at 37°C with 5% CO_2_. After washing with HBSS, fluorescence at 510/580 nm was measured every 2 min in a Flexstation 3 plate reader (Molecular Devices). Treatment additions were done after 3 min from starting the measurement.

### Viability Assay

LS8 cells were plated and treated with H_2_O_2_ (10 and 500 μM) for different time exposure (15 and 30 min, 1–24–48 h). Then, cells were loaded with Hoechst 350/461 nm (0.2 to 5 μg/mL; Thermo Fisher Scientific) and propidium iodide 493/636 nm (1 μM; Thermo Fisher Scientific). Images were taken using a Leica TCS SP5 II confocal microscope and edited using ImageJ (NIH).

### Cytofluorimetric Analysis

EO cells were isolated and suspended in FACS buffer (1% FBS in PBS) as previously described (Chen et al., 1998). Fluorescent PE anti-rat CD90/mouse CD90.1 (Thy-1.1) (1:500; BioLegend) was used for 30 min at 4°C in the dark to allow to distinguish between fibroblasts and ameloblasts. The analysis was performed by a flow cytofluorimetry (FACSAria IIu SORP cell sorter; BD) using FACSDiva software. Data for 10 K to 20 K events were collected.

### Immunocytochemistry

EO cells were isolated for immunohistochemical analysis and, after 24 h plating on 25 mm coverslips, fixed with 4% paraformaldehyde. Immunofluorescence staining was performed as follows: briefly, the cells were blocked and permeabilized with BSA 1%, Triton 0.1% in PBS for 5 min. After two washes in PBS, the following primary antibodies were used: anti-Ambn (1:500 dilution; SantaCruz sc-50534), and anti-AmelX (1:500 dilution; SantaCruz; sc-32892). After washing, detection was carried out using Alexa Fluor 488 (1:500 dilution; Life Technologies). Samples were embedded using Fluoromount mounting medium (Novus) containing DAPI. Images were taken using a Leica TCS SP5 II confocal microscope and edited using ImageJ (NIH).

### Transmission Electron Microscopy (TEM)

Cells were fixed with 1.25% (v/v) glutaraldehyde in 0.1 M sodium cacodylate at pH 7.4 for 2 h at room temperature, then conserved in 0.1 M sodium cacodylate pH 7.4. Slices of cells obtained after resin infiltration and polymerization were imaged on a Tecnai-20 electron microscope (Philips-FEI). Three independent samples per each cell type were prepared and labeled with a numerical code. Images from five different cells (more than 50 images) per sample were collected by the personnel of the electron microscopy facility (DeBio Imaging Facility, Department of Biology, University of Padova).

### Western Blot

EO total lysates were prepared in Laemli buffer (BioRad) and beta-mercaptoethanol (BioRad) and then loaded in 10% SDS-polyacrylamide resolving gels (BioRad). The membranes were saturated with fat-free milk 5% in TBS (Tris-HCl 50 mM, NaCl 150 mM, pH 7.5) Tween 0.1% for 1 h at room temperature and probed with antibodies against ETC complexes (Total OXPHOS rodent WB Antibody Cocktail, Abcam) and beta-Actin (Santa Cruz). Signals were amplified and visualized with horseradish peroxidase-conjugated secondary antibodies (BioRad) and detected by the ChemiDoc^TM^ XRS + with Image Lab^TM^ Software (BioRad). Western blots images were analyzed using the ImageJ software (NIH).

### Statistical Analysis

All statistical analyses of the data were done using Prism8 (GraphPad Software). A minimum of three independent experiments were performed. ANOVA or two-tailed unpaired Student’s tests were used to analyze statistical significance. Differences with *P*-values of < 0.05 were considered significant: ^∗^*P* < 0.05, ^∗∗^*P* < 0.01, ^∗∗∗^*P* < 0.001, and ^****^P < 0.0001. Results are shown as means ± SEM.

## Results

### Determination of Controls During Cell Isolation

To ensure that our dissections of enamel organ (EO) cells represented the secretory and maturation stages, we used the gene markers *Enam* and *Odam*, which are highly expressed in secretory and maturation stages, respectively (Supplementary Figure 1A) and we tested the expression of AmelX and Ambn in both the populations through immunofluorescence staining (Supplementary Figure 1B). Our data, shown in Supplementary Figure 1, confirmed that each cell population represents their respective developmental stage. The possible contamination of fibroblasts in the EO cell preparations was analyzed by cytofluorimetric analysis by labeling fibroblasts with the CD-90 marker. This analysis showed that fibroblast contamination in the EO cell dissections was small, with 10 and 8% found in our preparation for secretory and maturation, respectively (Supplementary Figure 1C). These data are in agreement with a previous report that indicated a 90% of ameloblasts by FACS using a very similar cell isolation method (Chen et al., 1998).

### Mitochondrial Biogenesis

**FIGURE 1:**
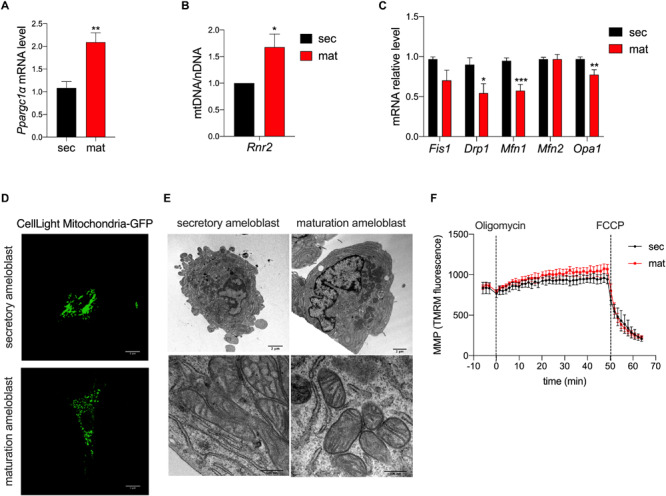
Mitochondrial morphology and biogenesis but not membrane potential differ in amelogenesis. **(A)** RT-qPCR analysis of peroxisome proliferator-activated receptor gamma coactivator 1-alpha (*Pparcg1*α), a marker for mitochondrial biogenesis revealed upregulation of its mRNA in maturation. **(B)** RT-qPCR analysis of *Rnr2* (16S ribosomal RNA), a marker for mitochondrial DNA, which was normalized to *Gapdh* as a reference for nuclear DNA, show an increase in maturation. **(C)** RT-qPCR analysis of mitochondrial pro-fission (*Fis1, Drp1*) and pro-fusion (*Mfn1, Mfn2, Opa1*) factors show a decrease in their mRNA expression in maturation cells. **(D)** Mitochondrial morphology assessment using CellLight Mito-GFP show increased mitochondrial fragmentation in maturation (scale bar = 1 μm). **(E)** Transmission Electron Microscopy (TEM) micrographs further show the range of mitochondria shape and size which are present in secretory (left panel) and maturation cells (right panel) (scale bar = 2 μm). **(A–C)** Data represent mean ± SEM from at least *n* = 3–6 independent experiments (*P < 0.05, **P < 0.005, ***P < 0.001, two tailed unpaired Student’s *t*-test). **(F)** Mitochondrial membrane potential (MMP) measured using TMRM show similar values in secretory and maturation EO cells.

To determine differences in mitochondrial biogenesis between the secretory and maturation stages, we analyzed the expression of PGC1α (*Pparcg1*α) by RT-qPCR. *Pparcg1*α mRNA level was two-fold higher in the maturation stage than in the secretory EO cells (Figure 1A). We also determined the ratio of mtDNA to nDNA using *Rnr2* as a mtDNA gene marker which was compared to the *Gapdh* as a marker for nDNA. The mtDNA/nDNA ratio was higher in maturation EO cells (Figure 1B). These results suggest an increase in mitochondrial mass from the secretory to the maturation stage.

To deepen the knowledge on mitochondrial dynamics, we evaluated the mRNA expression levels of gene markers for the fission process, mitochondrial fission 1 protein (*Fis1*), dynamin related protein 1 (*Drp1*), the fusion process via Mitofusin 1/2 (*Mfn1/2*), and optic atrophy 1 (*Opa1*). We found that *Drp1*, *Mfn1* and *Opa1* mRNA levels were lower in the maturation EO cells, suggesting that mitochondrial fusion is decreased at that stage (Figure 1C). To further investigate this possibility, we loaded the cells with MitoGFP and analyzed them by confocal microscopy and TEM. Confocal microscopy showed higher mitochondrial fragmentation in maturation EO cells (Figure 1D), as also supported by TEM analysis. Secretory cells showed an enrichment in rough endoplasmic reticulum and less mitochondria whereas in maturation mitochondria appeared more rounded (Figure 1E).

### Mitochondrial Membrane Potential (MMP) Is Unchanged Across Stages

To determine possible differences in mitochondrial membrane potential (MMP) between the two stages, we loaded secretory and maturation EO cells with TMRM. Treatment with oligomycin A, an ATP-synthase blocker that hyperpolarizes mitochondria, showed no differences in MMP between secretory and maturation EO cells. Similarly, the addition of FCCP, which depolarizes the MMP, showed no differences between the two cell types (Figure 1F). These results indicate that MMP is unchanged across both stages.

### Mitochondria ETC Complexes and OXPHOS Are Upregulated in Maturation

**FIGURE 2:**
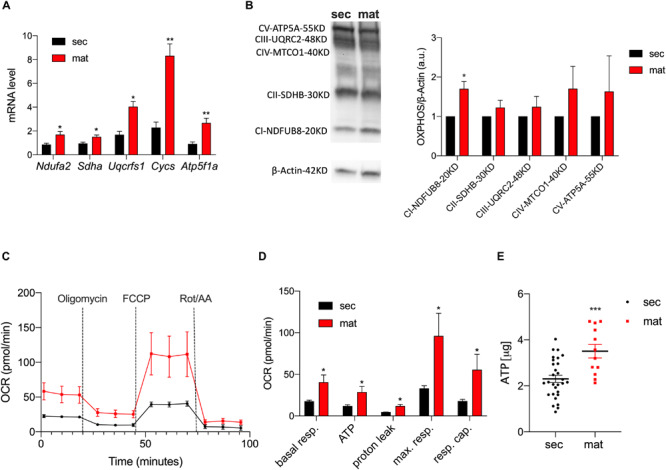
Mitochondrial respiration and ATP production are upregulated in maturation. **(A)** RT-qPCR analyses of components of the ETC subunits [NADH ubiquinone oxidoreductase subunit A2 (Complex I – Ndufa2), succinate dehydrogenase complex flavoprotein subunit A (Complex II – Sdha), ubiquinolcytochrome c reductase, Rieske iron-sulfur polypeptide 1 (Complex III – Uqcrfs1), cytochrome c, somatic (Cycs) ATP synthase F1 subunit alpha (Atp5f1a)]. **(B)** Western blot and ImageJ analyses of complexes CI-NDUFB8, CII-SDHB, CIII-UQCRC2, CIV-MTCO1, and CV-ATP5A show higher expression in maturation **(C)**. OCR traces (and respective quantification in **(D)** in secretory and maturation EO cells showed an overall increase in OXPHOS in the latter, with a significant increase in ATP production. **(E)** Higher ATP levels in maturation cells measured by luminescence. Data represent mean ± SEM from at least *n* = 3–6 (*P < 0.05, **P < 0.005, ***P < 0.001, two tailed unpaired Student’s *t*-test).

To highlight differences in the expression levels of the ETC complexes between the cell types, we measured the mRNA levels of the key components of the ETC by RT-qPCR (Figure 2A). The genes *Ndufa2*, *Sdha*, *Uqcrfs1*, *Cycs*, and *Atp5f1a* represent the components of complexes I, II, III, IV, and V of the ETC, respectively. We show that all of these were significantly upregulated in maturation EO cells. To further validate these data, we analyzed the protein levels of the ETC complexes by Western blot (Figure 2B). Complex I showed a stronger band in maturation, and all of the other complexes showed a trend toward higher levels in maturation EO cells overall (Figure 2B). To assess mitochondrial function at each stage, we analyzed differences in oxidative phosphorylation (OXPHOS) by quantitating the oxygen consumption rate (OCR) using the Seahorse Flux Analyzer to measure key aspects of cellular metabolism. We show that basal mitochondrial respiration, ATP production and spare and maximal capacity were all significantly higher in maturation EO cells (*P* < 0.05) (Figures 2C,D). In agreement with these results, ATP levels measured by luminescence showed higher total ATP levels in the maturation stage (Figure 2E). Combined, these data suggest that maturation EO cells experience an upregulation of OXPHOS, indicative of an increase in energy demand.

### ROS Are Elevated in Maturation

**FIGURE 3:**
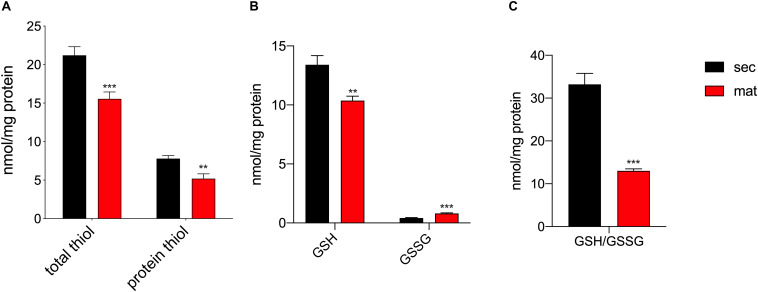
Maturation EO cells show increased oxidation. **(A)** Maturation stage EO cells show decreased total thiol and thiol protein levels, **(B)** decreased GSH and increased GSSG. **(C)** The GSH/GSSG ratio is significantly lower in maturation, indicating increased oxidation. Data represent mean ± SEM from at least *n* = 3–6 (*P < 0.05, **P < 0.005, ***P < 0.001, two tailed unpaired Student’s *t-*test).

It was previously suggested that the redox status and thiol modifications were relevant features in amelogenesis (Eckstein et al., 2017), although such differences across the stages had not been directly measured. The total amount of reduced thiols, as well as, the protein reduced thiol amounts were lower in the maturation stage (Figure 3A), suggesting the possibility of an enhanced oxidation of thiol groups associated with increased levels of post-translational modifications such as S-glutathionylation, as previously suggested (Eckstein et al., 2017). In addition, the upregulation of the mitochondrial complex I and III can be indicative of enhanced ROS production, as has been reported elsewhere (Schmidt et al., 1995, 1996; Kushnareva et al., 2002; Chen et al., 2003; Marinho et al., 2014; Booth et al., 2016). The different expression of these complexes in secretory and maturation EO cells shown above prompted us to analyze changes in ROS. We measured the levels of glutathione (GSH) levels, the most abundant antioxidant molecule in cells, as well as its oxidized form (GSSG), that is increased in conditions of elevated ROS (Griffith and Meister, 1985) to obtain the GSH/GSSG ratio, as this is an efficient parameter to assess changes in cellular redox state (Townsend et al., 2008; Zhang et al., 2018). Maturation EO cells showed lower GSH and increased GSSG than the secretory EO cells (Figure 3B). Consequently, the GSH/GSSG ratio was lower in maturation cells (Figure 3C), confirming an alteration in the redox state of these cells.

### ROS Modulate the Expression of EMPs

**FIGURE 4:**
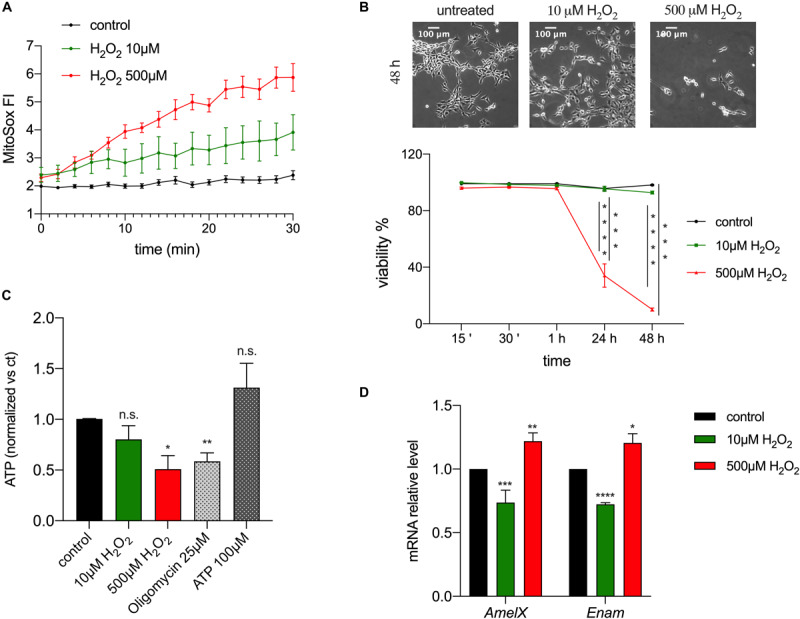
ROS modulates enamel gene expression. **(A)** H_2_O_2_ treatment of LS8 cells elicited a dose dependent enhancement of the MitoSOX fluorescence. **(B)** LS8 cells viability was significantly decreased upon treatment with H_2_O_2_ 500 μM, but not 10 μM (scale bar = 100 μm). **(C)** ATP content decreased when LS8 cells were exposed to 500 μM of H_2_O_2_ after 15 min. **(D)** LS8 cells exposed to both H_2_O_2_ concentrations (10 and 500 μM) show altered expression of *AmelX* and *Enam* mRNA levels. Data represent mean ± SEM from a minimum of three independent experiments (*P < 0.05, **P < 0.005, ***P < 0.0001, two tailed unpaired Student’s *t*-test).

The observed changes in redox in enamel cells suggested the possibility that ROS could act as a physiological modulator of cellular function, given that changes in ROS levels can accompany oxidative phosphorylation and that they have been associated with several signaling pathways, such as differentiation (Sauer et al., 2001), mitogenesis, senescence (Bladier et al., 1997), vascularization, inflammation and apoptosis (Reth, 2002; Valko et al., 2007). To address if ROS are involved in enamel formation, specifically by affecting expression of EMPs, alterations of ATP or cell viability, we used the ameloblast cell line LS8, one of the most commonly used enamel cell lines (Chen et al., 1992). We stimulated these cells with H_2_O_2_ to induce an increase in intracellular ROS. We used two concentrations of H_2_O_2_ (10 and 500 μM) as these are known to have an effect in cell signaling and Ca^2+^ homeostasis (Bogeski et al., 2010; Joseph et al., 2018). As expected, MitoSOX-loaded LS8 cells showed increased ROS levels when exposed to 10 and 500 μM of H_2_O_2_ (Figure 4A). To determine if H_2_O_2_ affected cell viability we monitored LS8 cells exposed to H_2_O_2_ over 48 h. LS8 cells were unaffected at the lower concentration of H_2_O_2_ (10 μM), but a concentration of 500 μM of H_2_O_2_ induced cell death (Figure 4B). To determine if H_2_O_2_ (10, 500 μM) affected cellular metabolism, we measured ATP levels after 15 min of treatment, as this time period had no effect on cell viability. We showed a significant decrease in ATP when cells were exposed to 500 μM of H_2_O_2_ but not with 10 μM. Extracellular ATP (100 μM) and oligomycin (25 μM) (see also Supplementary Figure 2) were used as controls (Figure 4C).

To assess if the increase in ROS induced by H_2_O_2_ played a signaling role, we measured the expression of the EMPs genes *AmelX* and *Enam* in LS8 cells exposed to H_2_O_2_ (10, 500 μM) for 15 min by RT-qPCR. This time point was chosen because it increased mitochondrial ROS (Figure 4A) and decreased ATP levels without causing cell death (Figure 4B). Treatment with low H_2_O_2_ (10 μM) concentration elicited a significant decrease in the expression of *AmelX* and *Enam*, whereas the opposite was found at high concentrations of H_2_O_2_ (500 μM) (Figure 4D). These results suggest either a direct role of H_2_O_2_ as a second messenger altering enamel gene expression, or an indirect effect via a modification in ATP levels. To test the latter, we measured the expression of *AmelX* and *Enam* by RT-qPCR after treating the cells with oligomycin (1, 25 μM) for 15 min to alter ATP levels (Neubert and Lehninger, 1962). At high concentration, oligomycin (25 μM) induced a significant increase in the expression of both genes but no significant differences were observed at the lower oligomycin (1 μM) concentration (Supplementary Figure 3). Together, these data indicate that a stimulus of low [H_2_O_2_] increased ROS and decreased the expression of enamel genes without affecting ATP levels. By contrast, while high [H_2_O_2_] also raised ROS levels, it resulted in significantly decreased ATP with upregulation of enamel gene expression. Based on these results, we suggest that moderate ROS changes are likely modulators of gene expression in enamel cells without eliciting changes in ATP.

## Discussion

The number and localization of mitochondria differ between secretory and maturation stage ameloblasts (Kallenbach, 1970; Assis et al., 2003; Eckstein et al., 2018), suggesting a potential difference in their function (Elwood and Bernstein, 1968; Jessen, 1968; Assis et al., 2003; Eckstein et al., 2018). We have analyzed basic physiological parameters of mitochondria in secretory and maturation stages of amelogenesis, focusing on mitochondrial biogenesis and respiration, as well as, outputs related to mitochondrial function such as the redox status. Results presented here show that mitochondrial function differs in secretory and maturation stage EO cells.

PGC1α is involved in regulating several mitochondrial pathways by its interaction with nuclear respiratory factor 1 and 2 (Nrf1, Nrf2) and also estrogen-related receptor alpha (ERRα) (Wu et al., 1999; Mootha et al., 2004; Scarpulla, 2008; Handschin, 2009). The trans-activation of these transcription factors may induce mitochondrial biogenesis through mtDNA transcription/replication (Schreiber et al., 2004; Scarpulla, 2008), fatty acid oxidation, transcription of components of the respiratory chain and the mitochondrial oxidative function (Wu et al., 1999; Mootha et al., 2004). *Pprgc1*α mRNA levels were upregulated in maturation EO cells, suggesting an increase mitochondrial biogenesis (Figure 1A). The mtDNA/nDNA ratio using *Rnr2* (16s ribosomal RNA), one of the most commonly used mitochondrial gene markers to quantify mtDNA (Gadaleta et al., 1989; Carabelli et al., 2011; Quiros et al., 2017), was higher in the maturation stage (Figure 1B), further supporting the notion of increased mitochondrial mass in maturation enamel cells. These data are in agreement with previous reports in which the volume and the surface of mitochondria were measured in secretory and maturation stage ameloblasts by light and electron microscopy (Kallenbach, 1970; Assis et al., 2003).

A key feature of mitochondria is that they are highly dynamic organelles undergoing fission and fusion to sustain their maintenance (Scorrano, 2013; Wai and Langer, 2016; Giacomello et al., 2020). Fission is required to create new mitochondria and to prime mitophagy (Rodger et al., 2018), while fusion attenuates stress by mixing the contents of potentially damaged mitochondria (Youle and van der Bliek, 2012; Liesa and Shirihai, 2013; Wai and Langer, 2016). Both processes are regulated by complex protein machineries that include the pro-fusion proteins *Mfn1, Mfn2* and *Opa1*, and the pro-fission factors *Fis1* and *Drp1* (Youle and van der Bliek, 2012; Liesa and Shirihai, 2013; Wai and Langer, 2016). With the exception of *Fis1* and *Mfn2*, mRNA levels of fusion and fission genes were downregulated in maturation suggesting an attenuation of these functions.

The shape of mitochondria in the maturation stage identified in our study appears more rounded than in the secretory stage cells (Figures 1D,E), consistent with observations made by Jessen (1968) and Josephsen and Fejerskov (1977). This might have implications for mitochondrial motility as round and smaller mitochondria are able to move more easily (Campello et al., 2006) than other shapes.

Mitochondria fragmentation is commonly linked to mitophagy (Rodger et al., 2018), an elaborated process involving distinct pathways from mitochondrial biogenesis. Mitophagy mediates mitochondria turn-over following mitochondrial damage or age (Narendra et al., 2008; Twig et al., 2008; Ashrafi and Schwarz, 2013), and it is defined as programmed-mitophagy in developmental and physiological contexts (Schweers et al., 2007; Ney, 2015). When mitochondria undergo mitophagy, they become engulfed by double-membrane autophagosomes which then fuse to lysosomes to be degraded (Rodger et al., 2018). To the best of our knowledge, no published data are available on mitophagosomes in the enamel cells, but in our preliminary analysis of counts of mitophagosomes using our TEM micrographs of EO cells, we did not observe any significant differences between the two stages (data not shown), although this should be more carefully detailed in future studies. The similar values obtained here for MMP in secretory and maturation EO cells (Figure 1F) support the notion, although preliminary, that mitophagy is not a relevant feature in amelogenesis.

In maturation stage ameloblasts, mitochondria accumulate near the distal pole adjacent to the ruffled-border and thus show a different localization than in secretory stage cells (Jessen, 1968; Josephsen and Fejerskov, 1977; Assis et al., 2003). Our findings that maturation stage cells show shape changes with a more fragmented appearance, along with increased mitochondrial biogenesis markers, would suggest that these processes enable the displacement and accumulation of mitochondria near the distal pole of maturation ameloblasts. This mitochondrial accumulation may potentially contribute to the movement of ions as previously suggested (Elwood and Bernstein, 1968; Jessen, 1968; Eckstein et al., 2018).

Higher mitochondrial mass may in part explain the observed upregulation of ETC components in the maturation stage (Figures 2A,B). We found a significant increase in basal and maximal respiration, higher ATP content and increased respiratory capacity in maturation EO cells (Figures 2C,D). As noted, this increase in OXPHOS in the maturation stage may be associated with the higher number of mitochondria. However, the metabolic requirements of secretory and maturation stage cells may also differ and account for changes in ETC function. We and others have shown that ATP-dependent pumps (SERCA-sarco/endoplasmic reticulum Ca^2+^-ATPase), H^+^-ATPases and channels (e.g., CFTR-cystic fibrosis transmembrane conductance regulator), are upregulated during maturation (Lacruz et al., 2012a). SERCA pumps are dedicated to the translocation of cytosolic Ca^2+^ into the lumen of the endoplasmic reticulum (ER) (Chen et al., 2019), whereas H^+^-ATPases likely facilitate the outward exchange of H^+^ that are generated either via the ETC, or through the activity of carbonic anhydrases (CA) of which at least the cytosolic CA2 is upregulated in maturation (Lacruz et al., 2012a). The increased activity of proteins requiring ATP in the maturation stage thus necessitates an increased availability of ATP. Without these critical functions performed by ATPases and CFTR, there would be an impairment to the development of healthy enamel. Therefore, a rise in energy demand in maturation must be met by a rise in ATP production that is matched by higher rates of electron transfer by the ETC. We suggest that the higher levels of OXPHOS in maturation fulfills the increased metabolic needs of ATP-dependent pumps and exchangers. Other physiological functions such as the recreation of the ruffled-border is also likely requiring a higher metabolic output. Previous reports support our hypotheses by showing increased cytochrome C oxidase (complex IV) levels in the maturation stage (Ohshima et al., 1998).

The generation of ROS is closely associated with the mitochondrial oxidative metabolism that releases ROS as a byproduct (Trinei et al., 2013). The increased in OXPHOS reported here in maturation cells is paralleled by differences in the redox status of secretory and maturation enamel cells. We investigated the GSH/GSSG ratio as an indicator of the redox environment in cells (Ballatori et al., 2009). Our results showed a reduction in the GSH/GSSG ratio in the maturation stage, indicative of increased oxidation (Figures 3B,C). We had previously argued that changes in redox in enamel cells might be physiological in nature (Eckstein et al., 2018). Accumulated evidence has shown that ROS plays a central role as a secondary messenger with the ability to modify proteins (Reth, 2002; Townsend, 2007; Zhang et al., 2018). In enamel cells, we proposed that S-glutathionylation could be important for signaling via the modification of thiols in proteins (Eckstein et al., 2017, 2018). We demonstrated this in the ameloblasts cell line, LS8 cells, where attenuation of GSH synthesis led to changes in SERCA function (Eckstein et al., 2019). Here we show that primary enamel organ cells from the maturation stage have a significant decrease in thiols, suggesting a likely increase in S-glutathionylation (Grek et al., 2013). This interpretation is in keeping with the decrease in GSH, limiting the ROS scavenging capacity of enamel cells in maturation.

To further investigate if ROS play a role in enamel formation, we measured changes in the expression of EMP genes essential for this process. ROS stimulation by H_2_O_2_, the main ROS metabolite involved in redox suitable to study physiological changes *in vitro* (Stone and Yang, 2006; Sies, 2017), elicited changes in the expression of *AmelX* and *Enam* mRNA level after 15 min of treatment (Figure 4D). To address if ATP levels influenced the expression of EMPs, we measured *AmelX* and *Enam* mRNA in LS8 cells treated with oligomycin as this is known to affect ATP levels (Neubert and Lehninger, 1962). Exposure to oligomycin (25 μM) resulted in upregulated mRNA levels of both genes with a significant decrease in ATP (Supplementary Figures 2, 3). By contrast, treatment with 1 μM of oligomycin, which does not affect ATP (Supplementary Figure 2), showed no significant changes in gene expression (Supplementary Figure 3). Together, these data indicate that low ROS levels can stimulate changes in enamel gene expression (Figure 4D) and because this occurs without altering ATP (Figure 4C), it suggests that ROS may function as a second messenger in enamel cells. Because the expression of *AmelX* and *Enam* decrease in maturation *in vivo* (Lacruz et al., 2017), and because we have shown here that ROS levels are elevated in maturation, it is tempting to suggest that the decrease in the expression of these two genes associated with a moderate increase in ROS in LS8 cells might be mimicking the physiological changes observed *in vivo*. Although further analyses are required to address this in more detail, these data would point to a physiological role of ROS in maturation potentially associated with decreasing the expression of enamel genes.

In summary, we have shown that the two main stages in the development and mineralization of dental enamel differ in mitochondrial shape and function. Maturation stage cells showed more round-shaped mitochondria that could be potentially more mobile. Our findings also show an increase in OXPHOS with higher ATP production in the maturation stage, possibly to meet ATP demands by ATP requiring proteins. Finally, increased OXPHOS likely resulted in elevated ROS (Trinei et al., 2013) that depleted GSH content, oxidizing the cellular environment, suggesting that ROS may play a signaling role in enamel.

## Data Availability Statement

All datasets generated for this study are included in the article/Supplementary Material.

## Ethics Statement

The animal study was reviewed and approved by Institutional Animal Care and Use Committee (IACUC) IA16-00625.

## Author Contributions

VC, MG, DT, and RL designed the study. All authors collected and analyzed data. VC, MG, DT, and RL wrote the manuscript.

## Conflict of Interest

The authors declare that the research was conducted in the absence of any commercial or financial relationships that could be construed as a potential conflict of interest.
