# Supplementary material for: Mitochondrial Function in Enamel Development
Source: Front Physiol. 2020 May 29;11:538. doi: 10.3389/fphys.2020.00538 (PMC7274036; doi:10.3389/fphys.2020.00538)
Supplement: Supplementary file 1 [file Data_Sheet_1.pdf]

SUPPLEMENTARY FIGURE CAPTIONS

Supplementary Figure 1. Secretory and maturation stage sample purity was assessed upon different techniques. (**A**) *Enam* (marker for secretory stage) and *Odam* (Odam) (marker for maturation stage) mRNA levels were quantified by RT-qPCR, confirming the purity of isolation of both stages. (**B**) Immunofluorescence staining was used to verify ameloblastin (Ambn) and amelogenin (AmelX) expression in secretory and maturation cells, which are specific ameloblast proteins (scalebar = 1 μm). (**C**) The proportion of positive cells for CD90 fibroblast marker was evaluated by cytofluorimetric analyses: our cell isolation procedure yielded 90% and 92% pure cultures (in secretory and maturation cell isolation respectively). Data represent mean ± SEM, from a minimum of 3 independent experiments. (*P < 0.05, **P < 0.005, ***P < 0.001, 2 - tailed unpaired Student's t test).

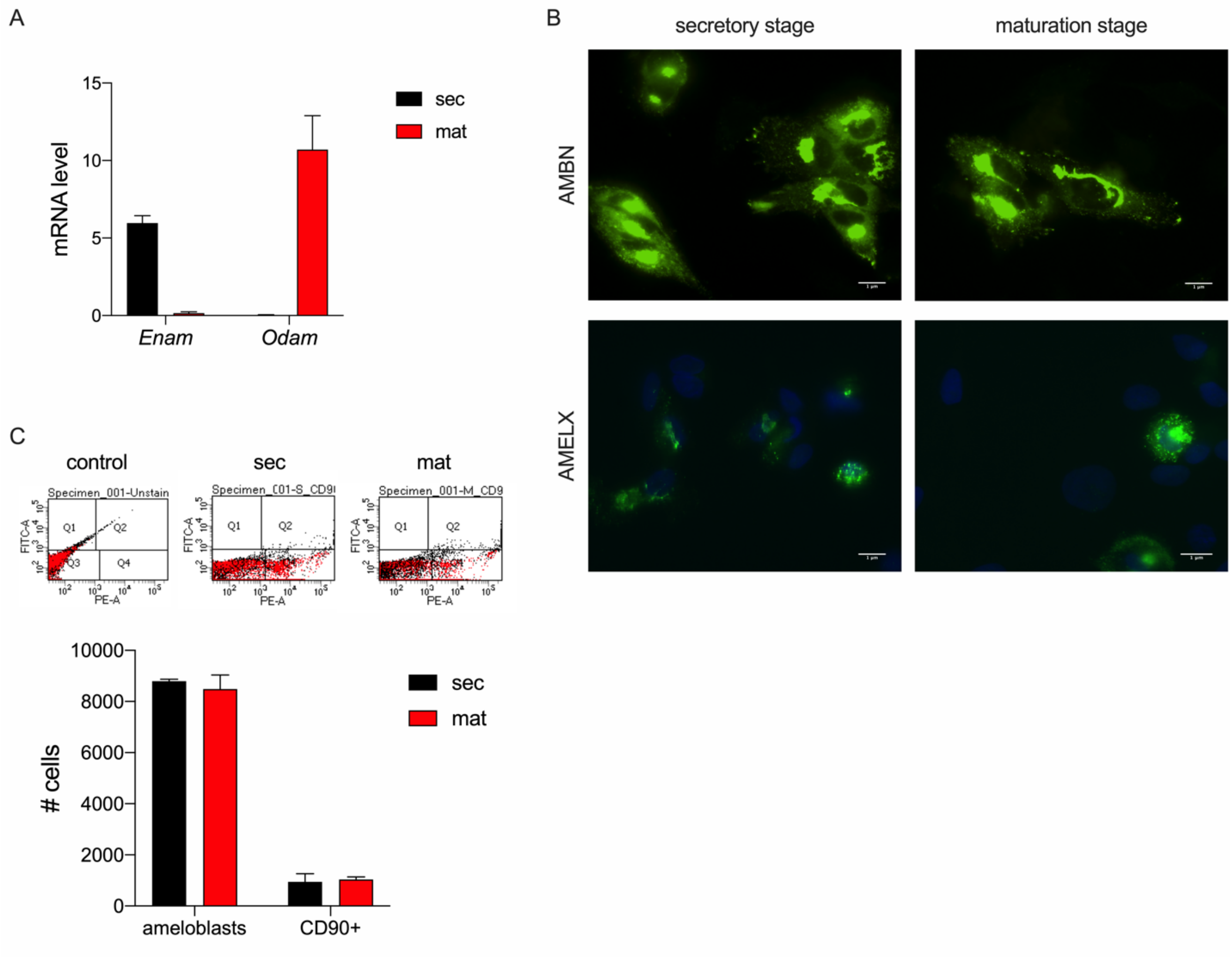

Supplementary Figure 2. Oligomycin decreases ATP levels in LS8 cells at high concentration. ATP content was decreased only when LS8 cells were exposed 15 min to 25 µM of oligomycin. Data represent mean ± SEM of 4 independent experiments. (*P < 0.05, 2 - tailed unpaired Student's t test).

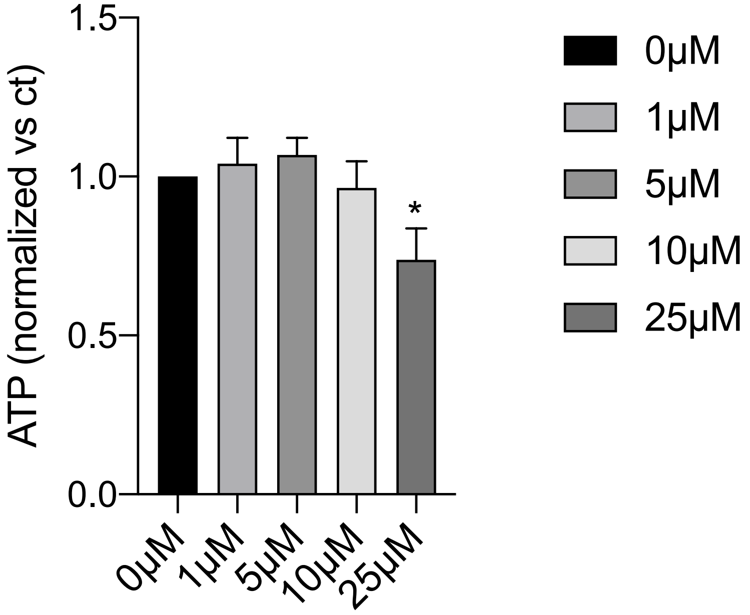

Supplementary Figure 3. Modulation of ATP by oligomycin alters enamel gene expression. LS8 cells were exposed for 15 min to oligomycin (1 and 25 µM) to detect changes in EMPs mRNA level. Only oligomycin (25 µM) elicited a significant increase in *AmelX* and *Enam* mRNA levels. Data represent mean ± SEM of 4 independent experiments. (*P < 0.05, **P < 0.005, 2 - tailed unpaired Student's t test).

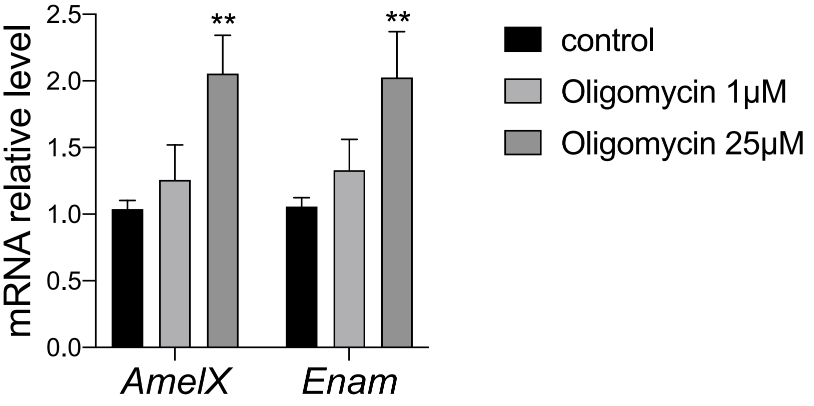

Supplementary Table 1. Primer sequences used for RT-qPCR.

Gene	**Forward Sequence**	**Reverse Sequence**	**Animal specificity**
Gapdh	CTGGAGAAACCTGCCAAGTA	TGTTGCTGTAGCCGTATTCA	mouse
AmelX	GCCGTATCCTTCCTATGGTT	GATGTTTGGCTGATGGTGTT	mouse
Enam	TATGGTCTTCCACCAAGGAA	TAGGCACACCATCTCCAAAT	mouse
Actin β	CACACTGTGCCCATCTATGA	CCGATAGTGATGACCTGACC	rat
Amelx	GCCGTATCCTTCTATGGTT	GATGTTTGGCTGATGGTGTT	rat
Atp5f1a	TAATGGCAAGCACGCTCTGA	TCAGCTTTGCATCCGACTGT	rat
Cyt C	GGTGTCGCCTCAAACCTATT	CGGGTGAGTCTTCTTGTTTCT	rat
Drp1	GGTGGAATTGGAGATGGTGGTCGA	TTCGTGCAACTGGAACTGGCACA	rat
Fis1	GTGCCTGGTTCGAAGCAAATAC	CATAATCCCGCTGCTCCTCTT	rat
Mfn1	ATCTTCGGCCAGTTACTGGAGTT	AGATCATCCTCGGTTGCTATCC	rat
Mfn2	CCTTGAAGACACCCACAGGAATA	CGCTGATTCCCCTGACCTT	rat
Ndufa2	AGGTACTGCGTGAGATTCGC	AAAAGCATAACGGGCCCAGA	rat
Odam	TTGACAGCTTTGTAGGCACA	TGACCTTCTGTTCTGGAAGC	rat
Sdha	AGCCTCAAGTTCGGGAAAGG	CAAGGTAAACCAGCCCGAGT	rat
Uqcrfs	GTTCCTGCTTCTGTTCGTTATTC	CTAGCCTCACTGCTCTCTTTAG	rat
**mtDNA/nDNA**			
Gapdh	GGAAAGACAGGTGTTTTGCA	AGGTCAGAGTGAGCAGGACA	rat
Rnr2	AGCTATTAATGGTTCGTTTGT	AGGAGGCTCCATTTCTCTTGT	rat
